# A Letter to the Inhabitants of Ceylon, on Vaccination

**Published:** 1839-04

**Authors:** 


Art. IV.—*A Letter to the Inhabitants of Ceylon, on the Advantages of Vaccination.* By J. Kinnis, m.d.—*Ceylon*, 1837. 8vo. pp. 28.

This is a well-written and interesting pamphlet on the subject of the past history of small-pox, and the present state of vaccination, in Ceylon. In a simple and affectionate address to the inhabitants of that island, Dr. Kinnis reminds them of the awful visitations which they have suffered from the small-pox; and, by tracing the history of each epidemic up to the year 1837, he satisfactorily demonstrates that their sufferings from this scourge have been entirely occasioned by their neglect of vaccination. This devoted island had, in former years, been the scene of frightful devastation from small-pox, which carried such terror into the minds of the natives, that, in several instances, upon the occurrence of the disease in a town or village, all the inhabitants fled, voluntarily incurring the miseries of hunger, and leaving their homes to be overrun by the wild beasts of the forest, rather than wait the attack of this still more fearful enemy.

Vaccination was first successfully performed in this island on the 11th of August, 1802; and its beneficial effects were soon manifest. By the end of 1802, small-pox was banished from the district of Hambuntotte; 1803 saw its extinction in the district of Colombo. By April, 1804, Galle and Matura were freed from it; Taffna, in 1806; and in January, 1806, five years and a half from the introduction of vaccination, the disease was extinct in the whole of the British possessions in Ceylon. A

few cases appeared at intervals between this time and 1810; but, from this date till 1819, the disease was not known. The consequence of this immunity was, that apathy and indifference to the means of their preservation took place of the vigilance and activity which their former sufferings had excited. Vaccination became gradually more and more neglected, until the enemy again suddenly appeared at their doors, and found the poor Ceylonese unprepared to resist him.

| | |
|---|---|
| "During the six months terminating on the 15th January, 1820, in the maritime districts alone | 5,451 |
| persons were ascertained to have had the disease, and | 1,745 |
| to have died, being nearly in the proportion of | 1 to 3 |
| or more exactly of | 10 to 31 |
| and, during the five months terminating on the same day, in the Kandian provinces | 2,423 |
| were admitted into the hospitals established for their accommodation, and | 1,200 |
| died; being nearly in the proportion of | 1 to 2 |
| "The total number of cases reported to government in the six months during which the disease chiefly prevailed, was | 7,874 |
| and the total number of deaths | 2,945 |
| being in the proportion of | 10 to 26 |

"This *awful punishment*, (says Dr. Kinnis,) inflicted alike on the guiltless child and its improvident parent, was brought on the people of Ceylon by their neglect of vaccination." (p. 10.)

This retribution for their neglect of the means of safety had the effect of once more rousing the inhabitants from their apathy, and vaccination was again very generally resorted to. From this time to the end of the year 1837, the small-pox has been epidemic in Ceylon four times; but in no instance to be compared in severity with the visitation of 1819.

Dr. Kinnis introduces a table which shows, in a striking manner, the coincidence of the epidemic with the gradual neglect of vaccination, and the stimulating influence of present terror in dispelling prejudices and rousing to renewed activity in seeking for future protection.

In answer to the objection raised against vaccination, that it frequently failed in proving a protection, Dr. Kinnis goes on to prove the position with which he commenced, "that, in the small proportion of cases in which small-pox occurs after vaccination, it assumes, in general, so mild a form as to excite little more apprehension than chicken-pox, and to be almost equally free from danger." During two epidemics, 737 cases of small-pox were reported, of which 550 had no satisfactory marks of vaccination; and of these last 198, died, giving the proportion of 10 to 28; or more than 1 to 3: while, of the remaining 187, who had satisfactory marks of vaccination, only 3 died, giving the proportion of 10 to 623, or 1 to 62.

We have been more desirous of noticing this small but valuable defence of vaccination, because the subject is one which excites at the present moment so deservedly strong an interest, from the very general prevalence of small-pox. Although we lament that the fond anticipations of the great Jenner have not been as yet realized, we entertain no doubt whatever of the efficacy of his invaluable discovery; we would even go as far as the immortal discoverer himself in our anticipations of success; but then we must extend our view to the time when vaccination and its merits shall receive that attention in this country which is undoubtedly its due; when the government shall give the weight of its authority to a plan for the general adoption of vaccination among all persons and classes; and when our own profession, as a scientific body, shall, by union and well-directed cooperation, bring the multitudes of facts and of opportunities for observation which they possess, to bear upon the many difficult and obscure questions which at present surround and check the beneficial progress of vaccination.

